# An online network tool for quality information to answer questions about occupational safety and health: usability and applicability

**DOI:** 10.1186/1472-6947-10-63

**Published:** 2010-10-22

**Authors:** Martijn DF Rhebergen, Carel TJ Hulshof, Annet F Lenderink, Frank JH van Dijk

**Affiliations:** 1 Academic Medical Centre Amsterdam/University of Amsterdam, Department: Coronel Institute of Occupational Health, P.O. Box: 22700, Meibergdreef 9, 1100 DE Amsterdam, The Netherlands; 2 Academic Medical Centre Amsterdam/University of Amsterdam, Department: Dutch Centre for Occupational Diseases, P.O. Box: 22660, Tafelbergweg 51, 1100 DD, Amsterdam, The Netherlands

## Abstract

**Background:**

Common information facilities do not always provide the quality information needed to answer questions on health or health-related issues, such as Occupational Safety and Health (OSH) matters. Barriers may be the accessibility, quantity and readability of information. Online Question & Answer (Q&A) network tools, which link questioners directly to experts can overcome some of these barriers. When designing and testing online tools, assessing the usability and applicability is essential. Therefore, the purpose of this study is to assess the usability and applicability of a new online Q&A network tool for answers on OSH questions.

**Methods:**

We applied a cross-sectional usability test design. Eight occupational health experts and twelve potential questioners from the working population (workers) were purposively selected to include a variety of computer- and internet-experiences. During the test, participants were first observed while executing eight tasks that entailed important features of the tool. In addition, they were interviewed. Through task observations and interviews we assessed applicability, usability (effectiveness, efficiency and satisfaction) and facilitators and barriers in use.

**Results:**

Most features were usable, though several could be improved. Most tasks were executed effectively. Some tasks, for example searching stored questions in categories, were not executed efficiently and participants were less satisfied with the corresponding features. Participants' recommendations led to improvements. The tool was found mostly applicable for additional information, to observe new OSH trends and to improve contact between OSH experts and workers. Hosting and support by a trustworthy professional organization, effective implementation campaigns, timely answering and anonymity were seen as important use requirements.

**Conclusions:**

This network tool is a promising new strategy for offering company workers high quality information to answer OSH questions. Q&A network tools can be an addition to existing information facilities in the field of OSH, but also to other healthcare fields struggling with how to answer questions from people in practice with high quality information. In the near future, we will focus on the use of the tool and its effects on information and knowledge dissemination.

## Background

Many people have questions on health or health-related issues, such as occupational health and safety (OSH) matters[1-3]. Although people in the Dutch working population have the legal and moral right to high quality answers, probably more than one million OSH questions remain un- or incompletely answered annually[3]. In theory, a knowledge infrastructure should provide high quality information (i.e. usable and evidence-based) to answer these questions through several facilities: information knowledge products, such as fact sheets or guidelines for practice; expert advice, such as from occupational physicians; and education and training by the company or as part of vocational training[4]. Clearly, the OSH infrastructure needs new or improved facilities or strategies that account for the barriers questioners in the working population experience in finding and using high quality information.

Specific research on the effectiveness of particular facilities or strategies for answering OSH questions of workers is absent. Almost all research on strategies to answer OSH questions is oriented towards professionals and the use of evidence-based practice strategies (EBP)[5,6]. Although the EBP strategy has been proved effective for OSH professionals, this strategy is not logical for the working population, as this strategy is time consuming and workers are not familiar with the terminology used in professional and scientific publications. Therefore, other strategies or facilities would probably be more suitable[1,7-9]. First, information and knowledge products are often numerous, free of charge and easily accessible, but these are not always specific or updated, and quality is regularly lacking. Second, OSH experts can provide high quality, and tailored answers quickly but are often not easily accessible or free of charge. Finally, education and training facilities could provide or support the finding of high quality answers, but they are time consuming and sometimes expensive. An interesting new strategy could be to combine useful elements of existing facilities through providing an online link between the person who is searching for information and an expert who provides tailored, high quality answers, possibly without charge.

Today, web-tools, such as patient forums, social networks (e.g., Facebook, LinkedIn) and Question and Answer (Q&A) network technologies, can establish such links. With these tools, an easily accessible network of experts answering questions can be created. Online Q&A network tools seem particularly promising for communication, information exchange, information storage and information retrieval[10,11]. The technology has already been applied in some large knowledge-intensive organisations, such as Philips and ABN AMRO Bank[10]. For these organisations, it is essential that experts easily find each other to exchange specialised information and knowledge. With a Q&A network tool, users may find a specific expert for their specific health or health-related problem and ask their question directly to that expert. The tool facilitates this process by sending an e-mail notification to the expert (when questioned) and the questioner (when answered). Questions and answers are stored in a searchable database for public re-use. To avoid privacy issues, a moderator can remove privacy-sensible information from the question or answer or prevent public access to the database before publication.

Although several models have described the process of designing and testing tools to suit the purpose of an intended new setting or context, many of these models included user-developer interactions[12]. When developing interactive information tools, the user-centred design of the International Organisation of Standardisation (ISO) is often applied (ISO 13407)[13]. A fundamental concept in this design process is usability[13]. Usability is defined as "the extent to which a product can be used by specified users to achieve specified goals with effectiveness, efficiency and satisfaction in a specified context of use (ISO 9241-100; 2009)"[14]. Usability is associated with high website satisfaction, use[15] and loyalty[16]. In addition to usability, we believe the perceived applicability of a new information tool is important for future use. In this study, we defined applicability as the perceived capability of a new tool to provide quality information to specific target groups under specific user conditions. Therefore, assessing both usability and applicability during the development of an online Q&A network tool is important.

In this study, the usability and applicability of the prototype of the online Q&A network tool ArboAntwoord http://www.arboantwoord.com was tested. This study is the first in a series of studies on the added value of the ArboAntwoord website within a given OSH infrastructure. The tool was created for all workers in every industry and sector who encounter difficulties in finding quality answers for their OSH questions. The website was launched through small-scale campaigns in which several articles were presented in national OSH magazines and websites. At launch in October 2008, 71 national experts in the field of OSH committed to the project and started answering all types of OSH questions from workers. The question topics were diverse, ranging from possible health risks of working with specific chemicals to return-to-work interventions for women with breast cancer and from work-climate law and regulations to safety solutions for working alone in small closed spaces. The aim of this study was to investigate the usability and applicability of an online Q&A network tool related to OSH for the intended user groups.

## Methods

### Q&A tool description

To develop the ArboAntwoord website, we used the existing software: XSanswers™ (Textinfo, Ede, The Netherlands). The homepage of the ArboAntwoord website initially comprised nine main categories that represent leading OSH topics (see Additional file 1: TIFF file - Screenshot webpage Select category - Search function). All main categories contained several subcategories. ArboAntwoord offers users two options for asking a question. The first is to formulate the question directly in the designated text field on the homepage, and the second is to use the button "ask your question" that is presented in all subcategories. Both possibilities will lead to a webpage in which the question must be given a title and the questioner must prohibit or authorise the publication of the question (see Additional file 2: TIFF file - Screenshot webpage Add title - Add question - Authorise publication). The last step in asking a question is the selection of an expert. Experts are registered in the subcategories that correspond with their expertise. Expert selection can be based on the appreciation offered by earlier questioners and on mean reaction time to previously answered questions (see Additional file 3: TIFF file - Screenshot webpage Select expert(s) - Button to send question - Expert reaction time and appreciation). A "send question to the expert" button is provided to automatically notify the selected expert about an asked questions. Subsequently, the selected expert will receive an e-mail notification with a direct hyperlink to the question. Experts answer questions in a main text field and can add an attachment when desired (see Additional file 4: TIFF file - Screenshot webpage Add answer in text field - Add attachment (optional) - Button to send answer). The answer is sent back to the questioner automatically with a "send answer to the questioner" button. All stored Q&A combinations are published and can be searched by other users when authorised by the questioner and the moderator (see Additional file 5 TIFF file - Screenshot webpage Hyperlink to view stored Q&A - Recent Q&A in (sub)category). When desired, experts can react to published questions and answers.

### Participant

Our intention was to discover 80% of all the unique, relatively rare usability problems (defined by being discovered by at least one third of the general population: p = 0.33). Therefore, in accordance with recommendations in literature, the minimal (sub)group size was set at four using as criterion: p(n)unique = 1 (1-p)^nsubj/ngroups^[17,18]. Subgroups were based on differences in internet and computer experience, and on both different user types. First, as computer and internet experience is an important factor influencing results in usability studies[18], the participants should represent a wide range of self-rated computer and internet experience. Therefore, the participants were categorised as computer and internet beginner, intermediate or expert based on two questions answered on a five-point Likert scale (range 1 - 5): 1) "How would you rate you computer experience?" and 2) "How would you rate your internet experience?" Very poor experience was rated as 1 point and excellent was rated as 5; thus, participants could score a maximum of 10 points. A participant with a summed score of 2-4 was defined as an internet and computer beginner, 5-7 as an intermediate and 8-10 as an expert. Second, as ArboAntwoord has two distinct user types, questioners from the working population (workers) and OSH experts, selection was also based on user type.

A worker was defined as an employer, a supervisor, an employee or a staff member with specific duties regarding OSH within a company or other work organisation. Through convenience sampling, we aimed to include 12 company workers with varying computer and internet experience from the Academic Medical Centre (AMC) in Amsterdam. The AMC is an academic hospital that comprises part of the University of Amsterdam (UvA). To identify company workers with computer and internet experience on the expert level, we approached workers in our Information and Communication Technology (ICT) department. For intermediates, we approached two health care departments, and for beginners, we addressed workers from the catering and transport service. Approached workers were given a short study introduction and were asked to rate their own computer and internet-experience. In total, we approached 20 workers. Three workers declined to participate due to time constraints, and one declined to participate because of a lack of interest. We excluded four workers due to saturation of the intermediate computer and internet experience groups. All participants received a gift coupon for 15 Euros for their participation.

An expert was defined as a person who has more than five years of experience working at national or international level with specific expertise in the field of OSH and who shares knowledge through publishing articles or participation in expert groups or boards. Experts were either scientific experts or practice experts. The experts represented a wide range of professional disciplines, such as occupational physicians, occupational hygienists, occupational safety workers, human movement scientists, health scientists, psychologists, neuropsychologists, dermatologist, internists, lawyers and OSH law and regulations experts. The experts were selected from a group of the 71 OSH experts committed to answering worker questions through the ArboAntwoord website. We invited all 71 experts to participate in this study by email, and 31 experts responded. Subsequently, we approached these experts by phone, asked them to rate their internet and computer experience and invited them to participate. We stopped inviting experts when the subgroups were saturated. As the group of 31 experts contained only experts with internet and computer experience at the intermediate and expert levels, only eight experts from this group could be selected to participate in our study. Therefore, we approached an additional eight of the 40 remaining experts whose computer and internet experience we thought was at the beginner level. Again, we were not able to identify any beginners.

### Study design and outcomes

To study the usability and applicability of our Q&A tool, we developed a test utilising two methods of data collection often used in human-computer interaction studies: observations and interviews[19]. The test was based on a usability design test protocol[20-22]. Additionally, the participants were asked to think aloud during task execution[20]. To consider the interaction between the participant and the specific feature, the participants had to carry out some computer tasks. After each task, the participant was interviewed by MR about that specific task. The test was finished with a general interview. The study was approved by the Ethical Committee of the Academic Medical Centre.

All tasks corresponded with the most important features of the website. Tasks and interviews were first tested for clarity and readability with one person of both user groups. The following tasks were included for the worker group: register as a website user (Task 1); ask a question to an expert (Task 2); search a stored Q&A combination (an answer) by using the search function (Task 3); search a stored Q&A in the (sub)categories using a direct overview with recent questions or a hyperlink to an overview of all questions in that subcategory (Task 4); and solve a technical problem by consulting the moderator or the help function (Task 5). As the working population and experts make use of partly overlapping but also different website features, the tasks for the two groups differed. Experts executed Task 1 and 4 as well as performed three other tasks: register as an expert by selecting his/her area of expertise (Task 6); answer a (fictitious) question (Task 7); and add a supplementary answer to a stored Q&A combination (Task 8). All eight usability task descriptions are presented in Table 1.

**Table 1:** Descriptions of the applied usability tasks

Task	Task description
**Task** **1**	**Register as a website user** ArboAntwoord.com is a semi-closed website, which means that every user must register the first time he/she wants to login. For every subsequent website visit, a username and password are sufficient to enter. You can reach the website by typing the following link in your web browser: http://www.arboantwoord.com. The assignment is as follows: register yourself as a user.
**Task** **2**	**Ask a question to an expert** Imagine that you have encountered the following technical problem: you do not know how to ask your question privately or anonymously. What can you do? ArboAntwoord.com provides two possibilities: 1. you can make use of the "help function"; and 2. you can call or e-mail the website moderator. The assignment is twofold: 1. find the "help function" and find out how to ask a question privately or anonymously; and 2. what is the phone number and e-mail address of the website moderator?
**Task** **3**	**Solve a technical problem by consulting the help function or the moderator** Imagine that you are a hairdresser and often suffer from skin irritation; you have dry, red hands, with scaling and your hands itch. You have noticed these complaints disappear when you have taken some time off work. How can you possibly prevent these complaints in the future? The assignment is as follows: ask this question through submitting it in the corresponding category and subcategory.
**Task** **4**	**Search a stored Q&A in the (sub)categories using a direct overview with recent questions or a hyperlink to an overview of all questions in that subcategory** It would be inefficient for both the experts and questioners to ask and answer the same question more than once. Therefore, ArboAntwoord.com saves and stores questions and answers and makes them accessible to other users. One way of finding stored questions is by looking in the subcategories. There all "recent questions" asked in this subcategory are presented. We have stored the following question (and answer): "What type of tests should be included in an assessment for an asbestos removal worker?" The assignment is as follows: find this question (and the answer) by searching the corresponding categories.
**Task** **5**	**Search a stored Q&A combination (an answer) by using the search function** It would be inefficient for both the experts and questioners to ask and answer the same question more than once. Therefore, ArboAntwoord.com saves and stores questions and answers and makes them accessible to other users. Another way of finding stored questions and answers is to make use of a search tool with search terms (such as Google). We stored the following question (and answer): "What happens when an occupational physician reports an occupational disease to the Netherlands Centre for Occupational Diseases (NCOD)?" The assignment is as follows: find this question by using the search function (and search terms).
**Task** **6**	**Register as an expert by selecting his/her area of expertise** Imagine that you have heard about ArboAntwoord.com and want to register as an expert. The assignment is as follows: register yourself as an expert.
**Task** **7**	**Answer a (fictitious) question** Earlier today, I have sent you an e-mail with a hypothetical question. This message is the usual e-mail ArboAntwoord.com automatically sends to the expert to whom the questioner asked his question. The assignment is as follows: open this e-mail, make use of the hyperlink that leads to the question and answer it with an "imaginary" answer.
**Task** **8**	**Add a supplementary answer to a stored Q&A combination** Presumably, experts want to have oversight of the recent questions and answers in their area of expertise. When reading these questions, experts might want to add information to or even correct the answer. For this purpose, ArboAntwoord.com provides the opportunity to give an additional answer or reaction to recently answered questions. We stored the following question (and answer): "What are the risk factors for Occupational Hand Eczema?" The assignment is as follows: give an "imaginary" reaction to this question. You can locate the question through: → main category "work health risks" → subcategory "irritating substances" → (sub)subcategory "water and soap" → The first question in "recent questions".

Usability, consisting of effectiveness, efficiency and satisfaction, was defined according to ISO 92411-100[14]: *effectiveness *is the (accuracy and) completeness with which users achieve specified goals; *efficiency *is the resourses expended in relation to the accuracy and completeness with which users achieve goals; *satisfaction *is freedom of discomfort, and positive attitudes to the use of the product. Effectiveness and efficiency were assessed by task observations. In this study, a task was executed effectively when a participant completed the task and ineffectively when the task was not completed. We categorized task efficiency as follows: 1) Efficient (participant completes the task without problems or alternative pathways); 2) Partly efficient (participant completes the task with one or two problems or uses one or two alternative pathways); 3) Partly inefficient (participant completes the task with more than two problems or more than two alternative pathways); and 4) Inefficient (participant does not complete the task at all; this result also means not effective). To determine satisfaction, all participants were asked one question during the specific task interviews: How satisfied are you with this aspect of the website? Because of the small sample size, a three-point Likert scale was used: 1) Dissatisfied; 2) Neither satisfied nor dissatisfied; and 3) Satisfied. Lastly, information on *facilitators *and *barriers *in content, navigation, lay-out, use of language and possible *improvements *of the website features were collected by the following open-ended questions during the task interviews: What facilitators or barriers did you experience in the content, navigation, layout or used language of this feature? Do you have any suggestions for improvement?

*Applicability *was assessed by three open-ended questions in the general interview: 1) "Is this website, in your opinion, an applicable tool for obtaining information?"; 2) "For whom in particular is this website, in your opinion, applicable?" and 3) "What are, in your opinion, important requirements for this website in order to be used?" For questioners, the questions focused on the applicability of the tool for information on health or healthcare. The experts' questions focused on providing information on occupational health or healthcare.

### Setting

**Figure 1:**
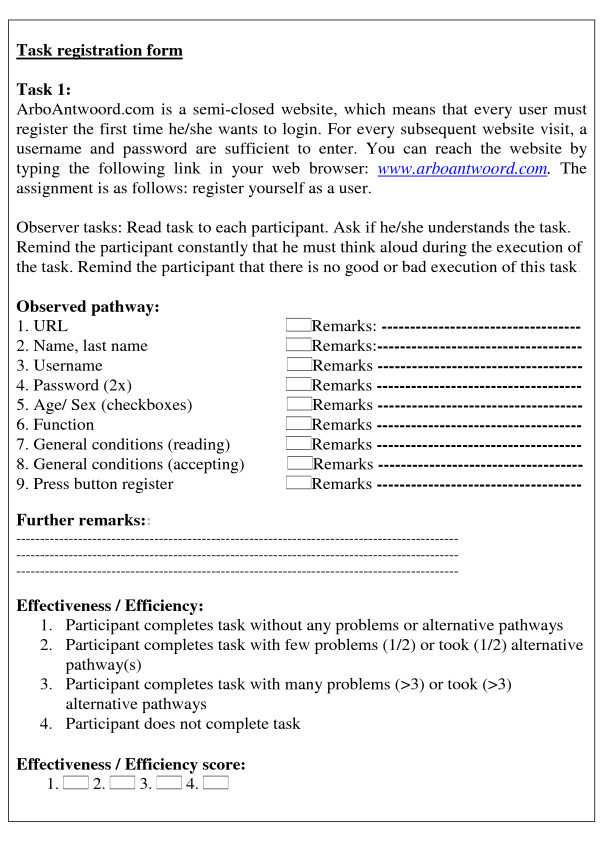
**Example of a usability task observation form (Task 1): register yourself as a website user**.

The test was conducted at the participants' own computer worksites on a desktop computer with speakers and internet connection. Before each task, the observer instructed the participant by reading the participant a short script of the tasks. The participants also received instruction forms with all the tasks to read along with the observer. The observer asked whether the participant understood the task. Subsequently, the participant was asked to perform the task while 'thinking aloud'. During the execution of tasks, the main researcher (MR) observed how the participants interacted with features of the tool. To define effectiveness and efficiency, MR observed and noted the pathways used on task-specific forms (Figure 1). The entire test and general interview were audio-taped, to increase reliability. Testing took approximately 1 hour and 15 minutes for each participant.

### Data analysis

All audio-taped interview data was analysed by employing descriptive analysis and content analysis of all transcripts[23], using MAXQDA software (VERBI Software, Marburg Germany, 2006). MR read all transcripts and extracted relevant statements, which were checked by another member of the research team (CH). Every relevant statement was coded according to a taxonomy that corresponded with the interview questions on content, navigation, lay-out, language and applicability. Statements that could not be coded to this taxonomy were (iteratively) discussed by MR and CH, and by consensus, new codes were created. Due to the small sample size of this study, the data presented in this paper are mainly descriptive.

## Results

### Participants

Eight experts and 12 possible questioners participated in the study. In Table 2, characteristics of the participants are summarised.

**Table 2:** Personal characteristics of the questioners and the experts

Group	Sex	Mean age (min - max)	Computer and internet experience	Education
*Experts*	7 male, 1 female	48.4 (41 - 59) years	4 medium, 4 high	8 high
*Questioners*	8 male, 4 female	31.9 (22 - 62) years	4 low, 4 medium, 4 high	4 high, 7 medium, 1 low

### Usability

#### Effectiveness

Most participants executed the tasks effectively; the majority of the tasks were completed as expected. Only Task 2 (asking a question) was not finished by two participants with no computer- and internet-experience (Table 3). One participant could not finish any of the five tasks (without help). This participant did not use a computer at work and only used Microsoft Office applications at home.

**Table 3:** Task usability results: effectiveness, efficiency (task observations) and satisfaction (task interviews)

Task/feature	N	Effectiveness	n	Efficiency	n	Satisfaction	n
*Task 1*	20	Effective	19	Efficient	11	Satisfied	17
*Register*				Partly efficient	8	Neither satisf./dissatisf.	2
				Partly inefficient	0	Dissatisfied	1
				Inefficient	1		
							
*Task 2*	12	Effective	10	Efficient	3	Satisfied	3
*Ask a question*				Partly efficient	5	Neither satisf./dissatisf.	7
				Partly inefficient	2	Dissatisfied	2
				Inefficient	2		
							
*Task 3*	12	Effective	11	Efficient	5	Satisfied	9
*Search answer by words in search function*				Partly efficient	4	Neither satisf./dissatisf.	1
				Partly inefficient	2	Dissatisfied	2
				Inefficient	1		
							
*Task 4*	20	Effective	19	Efficient	3	Satisfied	7
*Search answer by category*				Partly efficient	5	Neither satisf./dissatisf.	8
				Partly inefficient	11	Dissatisfied	5
				Inefficient	1		
							
*Task 5*	12	Effective	11	Efficient	3	Satisfied	7
*Technical help from moderator or help function*				Partly efficient	4	Neither satisf./dissatisf.	3
				Partly inefficient	4	Dissatisfied	2
				Inefficient	1		
							
*Task 6*	8	Effective	8	Efficient	3	Satisfied	3
*Register as expert*				Partly efficient	1	Neither satisf./dissatisf.	2
				Partly inefficient	4	Dissatisfied	3
				Inefficient	0		
							
*Task 7*	8	Effective	8	Efficient	6	Satisfied	6
*Answer a question*				Partly efficient	2	Neither satisf./dissatisf.	1
				Partly inefficient	0	Dissatisfied	1
				Inefficient	0		
							
*Task 8*	8	Effective	8	Efficient	5	Satisfied	6
*Add a supplementary answer*				Partly efficient	3	Neither satisf./dissatisf.	2
				Partly inefficient	0	Dissatisfied	0
				Inefficient	0		

Participants (N = 20) are questioners from the working population (N = 12) and experts (N = 8). Questioners executed Tasks 1, 2, 3, 4 and 5. Experts executed Tasks 1, 4, 6, 7 and 8.

#### Efficiency

The efficiency varied over the tasks (Table 3). Registration, search answers by words in the search function, answering a question and adding a supplementary answer to a stored Q&A combination were performed (partly) efficient by most participants. Other features, asking a question and technical help from the moderator or the help function, were executed (partly) inefficiently by some participants. Search by category and expert registration were performed (partly) inefficiently by most participants.

#### Satisfaction

Most participants were satisfied with the following features: register, search answers by words in search function, technical help from moderator or help function, answer a question and add a supplementary answer. The following features were classified as neither satisfied nor dissatisfied by most participants: asking a question, search answer by category and register as expert (Table 3).

#### Facilitators, barriers and improvements

Four features had insufficient usability: asking a question, search answer by category, technical help from moderator or help function and register as expert. These four features therefore deserve special attention. Essential statements about the barriers and (possible) improvements of these features made by the participants during the task interviews are presented below, and an overview of all statements is presented in an additional file (see Additional file 6: Text file - Overview of all statements about facilitators, barriers and improvements of all eight features mentioned by the participants during task interviews).

While asking a question, the participants stated that it was easy to navigate through a predefined pathway to a single end-point: press button to send a question to an expert (see Additional file 3: TIFF file - Screenshot webpage Select expert(s) - Button to send question - Expert reaction time and appreciation). However, this process could be improved by limiting the amount of scrolling and adding tracking (steps) for the current process. Next, participants stated that questioners should select experts themselves. The computer should not make the "best" choice based on ratings and answering speed of experts (see Additional file 3: TIFF file - Screenshot webpage Select expert(s) - Button to send question - Expert reaction time and appreciation).

Usability results showed that the participants encountered difficulties when searching stored answers in the (sub)categories. Participants stated that they experienced the categories as unclearly defined or illogical. To facilitate searching in the categories, they should be complete, logical and unambiguous, ordered alphabetically and/or chronologically in organ systems or risk factors and for different target groups. Consequently, we redesigned the categories in ten new main categories: 1) Health complaints caused by work; 2) Health and safety risks in work; 3) Working with health complaints; 4) Improving work conditions; 5) Coping with work disability; 6) Testing work demands; 7) Special groups of workers; 8) Branches, sectors, Industries; 9) OSH law and regulation; and 10) Other/remaining questions.

Solving a technical problem with the help function was not performed efficiently by all participants. In the help function, technical problems (subjects) are presented as hyperlinks to answers. The participants stated that the hyperlink to the help function itself was too difficult to find. Instead of being at the bottom of a webpage, the hyperlink to help should be placed in the header. The hyperlinks were formulated as a question. Participants stated that this was easy to use because it resembled Windows.

The experts encountered several difficulties in expert registration. First, they thought double registration first as a user and then as an expert (where they must indicate their area of expertise and ask for expert authorisation) on two different website locations was illogical. The experts suggested integrating them both. Second, the participants suggested making the registration process more transparent for new experts. They suggested to presenting the rules for expert participation and explaining the expert registration process.

### Applicability

The interviews showed that ArboAntwoord was regarded an applicable information tool by most questioners. The applicability results are summarised in Table 4. Easy access to experts was mentioned as an important advantage of ArboAntwoord. Nevertheless, half of all questioners reported preferring an additional face-to-face consult with a familiar expert (i.e. a general practitioner). A number of participants noted that ArboAntwoord was appropriate for non-urgent problems and additional information. The experts were of the opinion that the website was especially applicable for observing new OSH trends and for increasing the contact with people in practice. Some of the questioners considered the website mainly suitable for people with average or higher than average computer and internet experience. The experts thought that the website should be accessible only to semi-professionals in OSH fields as otherwise the number of incoming questions would be too high. Many questioners and experts stressed that the reliability of a website is increased by the hosting and support of a trustworthy organization and moderator. Finally, other important requirements mentioned by the participants were as follows: effective implementation or media campaigns, timely answering (<1 week), and anonymity.

**Table 4:** Applicability statements of questioners (N = 12) and experts (N = 8) in general interviews

	Questioners (N = 12)	N	Experts (N = 8)	n
*Applicability*	Applicable, however, in addition face-to-face consult with familiar expert: because of habit, reliability or confidence	6	Applicable for observing new OSH trends Applicable for increasing contact with practice	5 4
	Applicable because of easy access to expert(s)	4	Applicable because questioners from practice have easy access to expert(s), which is usually difficult	2
	Applicable for non-urgent problems	4	Answer more applicable when more experts answer	2
			Not applicable when lay-persons cannot describe context	2
*Target* *populations*	Computer and internet experience necessary	3	Questions only by semiprofessionals, otherwise too many questions	3
			Mainly for occupational physicians	2
*Important* *requirements*	High reliability through hosting and support by trustworthy professional organization and moderator	7	High reliability because of hosting and support by trustworthy professional organization and moderator	7
	Effective implementation or media campaigns to reach working population	5	Website must generate some income to support experts, moderator and technique	3
	Timely answers (< 1 week)	3		
	Anonymity of questioners	2		

Only statements mentioned two or more times are presented in this table.

## Discussion

The findings of this study showed that most features of our prototype Q&A network tool were usable, although some of them could be improved. The majority of the tasks were executed effectively, whereas task efficiency and satisfaction varied. Participants helped to identify various possibilities for improvement, including features such as the process of asking a question, searching for an answer by category, obtaining technical help from the moderator or help function and expert registration. As a result, in the revised version of ArboAntwoord, launched in October 2008, we limited the amount of scrolling and added tracking (steps) to the questioning process, allowed questioners to select experts themselves, redefined (sub)categories, moved the hyperlink to the help function in the header, registered experts ourselves and presented the rules for expert participation.

The results of our study further suggested that an online network tool is an applicable information tool for the OSH field. Some questioners preferred to consult a familiar expert in as well. The tool was stated to be applicable for non-urgent health problems and for gathering additional information. The experts stated that the system might assist in observing new OSH trends and might facilitate contact between questioners from the working population and experts. Hosting and support by a trustworthy professional organisation, anonymity, timely answers and effective promotion campaigns were mentioned as important requirements for use. Usability findings and participant remarks on online Q&A target groups indicate that online Q&A network tools are not applicable for people with no or only limited computer or internet experience. To provide OSH information to this sub-set of workers, asking a question directly or indirectly through a coordinator by telephone could be an alternative.

Little is known about the applicability and usability of Q&A tools and similar online networks for high quality information and knowledge in healthcare, although similar tools, such as tele-consulting systems and patient forums, have been discussed in the literature. Q&A tools are different in some respects (i.e. they include more or less extensive network features, self-selection of experts by questioners, e-mail notification and an easily accessible public database). Notwithstanding differences, comparison is useful. Marco et al.[24] studied an "ask-the-expert-service" of a consumer-oriented website on HIV-AIDS. Despite the fact that there was only one expert answering questions, the authors concluded that there was a great demand for online "ask-the-expert" services. This opinion shared by Umefjord et al.[25] who studied a similar service for enquiries related to health or diseases. These researchers found that an ask-the-expert-service was mostly used because of anonymity and convenience. Asking the questions and viewing the answers at a self-chosen time was highly appreciated. Other important reasons for use were to become better informed, to obtain a second opinion and to present embarrassing concerns and worries anonymously. Similar reasons for use were found by Himmel et al.[26], who studied an expert forum on infertility. The importance of a "second opinion" was also brought forward as a reason for seeking tele-advice by Eysenbach et al[27], who studied patients asking questions mainly in the field of dermatology (unsolicited e-mails sent to physicians). Marco et al.[24] stated that the facilitating conditions for the success of an ask-the-expert-service were anonymity, free access and timely answers. Massone et al.[28], who studied a non-commercial tele-consulting system in the field of dermatology, concluded that these systems are promising when they are non-commercial, discretionary, multilingual and open-access in nature. Important reasons for using an ask-the-expert-service are the easy access and the additional information or second opinion about specific health issues or interventions[24-27]. Both reasons were confirmed by several participants in our study. Other facilitating reasons for use, such as anonymity and timely answers, are also in accordance with the results in this evaluation.

A number of possible negative aspects of expert answers or online Q&A tools have been addressed in the literature as well[7,25,26]. Schaafsma et al.[29] showed that, with respect to occupational health issues, experts do not always provide valid answers when compared with evidence from the literature. That study also found that answers from the consulted experts that included references or sources in general were more valid than answers without such sources. Therefore, in addition to selecting experts on their knowledge, experts should be encouraged to add sources or references to their answers. Eysenbach et al.[7] warned that people could overuse ask-the-expert-services in their desperate search for additional information. Receiving too many questions can create a problem for the participating experts. The experts in our study also indicated their concern about receiving too many questions. To encourage expert participation, we provide experts with a 10 Euro incentive for each answer. In addition, we developed new features for ArboAntwoord through which experts can now do the following: 1) define the amount of questions they want to receive each month; 2) return non-relevant questions to the questioners; and 3) pass on questions to other, more suitable experts in the network. In this way, experts can regulate the amount of questions they receive. Furthermore, answers may be too complex for a questioner to understand [25,26]. Himmel et al.[26] warned of the possibility that answers may be superficial. Either way, in ArboAntwoord, we try to prevent this by adding a feature that allows questioners to ask an "additional question" in reaction to the answer of an expert. A questioner can ask for a clearer or more thorough explanation of the first answer. Another possible adverse aspect was mentioned by Eysenbach et al.[7]: many users are sending excessively personal details over the Internet. Preserving privacy is of paramount importance for these types of online Q&A tools. Therefore, we applied a Secured Sockets Layer (SSL) for ArboAntwoord. SSL is an encrypted protocol that secures communication through the Internet. All questioners ask their questions anonymously, so no personal information is published on the web. Finally, we created a feature by which questioners and the moderator have to authorise Q&A combinations to be published in the public database. The moderator screens all Q&A combinations on suitability before publication. The moderator can remove sensitive personal information in a Q&A combination before publication, omitting or changing localities, gender, age, occupational and/or medical details. The questioner and moderator can also choose not to publish the Q&A combination at all. We think that a hosting organisation should draw special attention to legal matters and privacy policy. A disclaimer is clearly a good start but is not sufficient.

The strength of our study lies in the user-centred design used to evaluate and improve important features of this new Q&A network tool for OSH before implementation. However, the study has several limitations as well. First, the sample was limited and unevenly distributed with respect to age and sex, which may lead to overestimation or underestimation in the study results[30]. In addition, the sample was not entirely representative for purposes of assessing applicability. It would have been better to recruit a larger sample from different settings, particular organisations or occupations, who actually had (answered) OSH questions. Second, the test took place in a field setting: the participants' workplace. Possible differences in this setting such as screen size, internet connection speed and keyboard features can result in dissimilar experiences and different research results. However, the advantage of a field test is that it represents the real life situation better than a laboratory experiment. A third limitation is the data collection method. Observation, for example, has advantages and disadvantages in comparison with methods such as video recordings. The investigator might miss some navigation paths, resulting in an overestimation of task efficiency. Moreover, the observer may somehow influence a participant. Sitting just behind a participant may create a feeling of being rushed, which may lead to mistakes. A usability laboratory can facilitate in more rigorous data collection. Next, evaluating a system that was also developed by the evaluators could raise a conflict of interest. For example, interviewees knew that we were developing a new information tool. This knowledge could have elicited gratifying responses. We tried to overcome this by creating an open atmosphere, in which participants were encouraged to find usability problems. Another limitation is the think-aloud protocol applied in this study. Thinking aloud during usability tests can facilitate finding problems, as it reflects the actual use of a feature rather than the participant's judgment[19,31]. Therefore, some authors have noticed that think-aloud interviews can impede the discovery of usability problems[32] and task performance[20].

## Conclusions

In conclusion, our online Q&A tool is a promising new strategy for providing company workers with high quality information to answer OSH questions. The revised version, launched after this study, addressed the concerns and usability problems that were raised in the test and the interviews. Our tool seems to be particularly applicable to the provision of additional information on non-urgent health and safety topics, and can possibly improve contact between questioners from companies and OSH experts. Hosting and support by a trustworthy professional organisation, anonymity, timely answering and effective promotion campaigns were identified as important requirements for use.

This study indicates that Q&A network tools can be an interesting addition to existing information facilities in the field of OSH and in other healthcare fields that are looking for new strategies to answer questions from people in practice (workers, patients, or professionals) with high quality information. Nonetheless, this study was just a first step in a larger evaluation of the Q&A tool ArboAntwoord. In the near future, we will study the actual value of this tool within a given OSH knowledge infrastructure. We will focus on the use of the tool, the answer quality and the effects on information and knowledge dissemination in general. We recommend research on the use and effects of Q&A tools in different contexts.

## Competing interests

The authors declare that they have no competing interests.

## Authors' contributions

MR, CH, FD and AL designed the study. MR and CH planned the analysis, collected data. MR and CH analysed data. MR, CH, FD and AL wrote the paper. All authors read and approved the final manuscript.

## Pre-publication history

The pre-publication history for this paper can be accessed here:

http://www.biomedcentral.com/1472-6947/10/63/prepub

## Supplementary Material

Additional file 1 **Select category - Search function**. Click here for file

Additional file 2 **Add question - Authorise publication**. Click here for file

Additional file 3 **Select expert(s) - Button to send question - Expert reaction time and appreciation**. Click here for file

Additional file 4 Add answer in text field - Add attachment (optional) - Button to send answer Click here for file

Additional file 5 **Hyperlink to view stored Q&A - Recent Q&A in (sub)category**. Click here for file

Additional file 6 **Overview of all statements about facilitators, barriers and improvements of all eight features mentioned by the participants during task interviews**. Participants (N = 20) are questioners from the working population (N = 12) and experts (N = 8). Questioners executed Tasks 1, 2, 3, 4 and 5. Experts executed Tasks 1, 4, 6, 7 and 8. Click here for file
