# Supplementary figures and images for: An online network tool for quality information to answer questions about occupational safety and health: usability and applicability
Source: BMC Med Inform Decis Mak. 2010 Oct 22;10:63. doi: 10.1186/1472-6947-10-63 (PMC2987966; doi:10.1186/1472-6947-10-63)

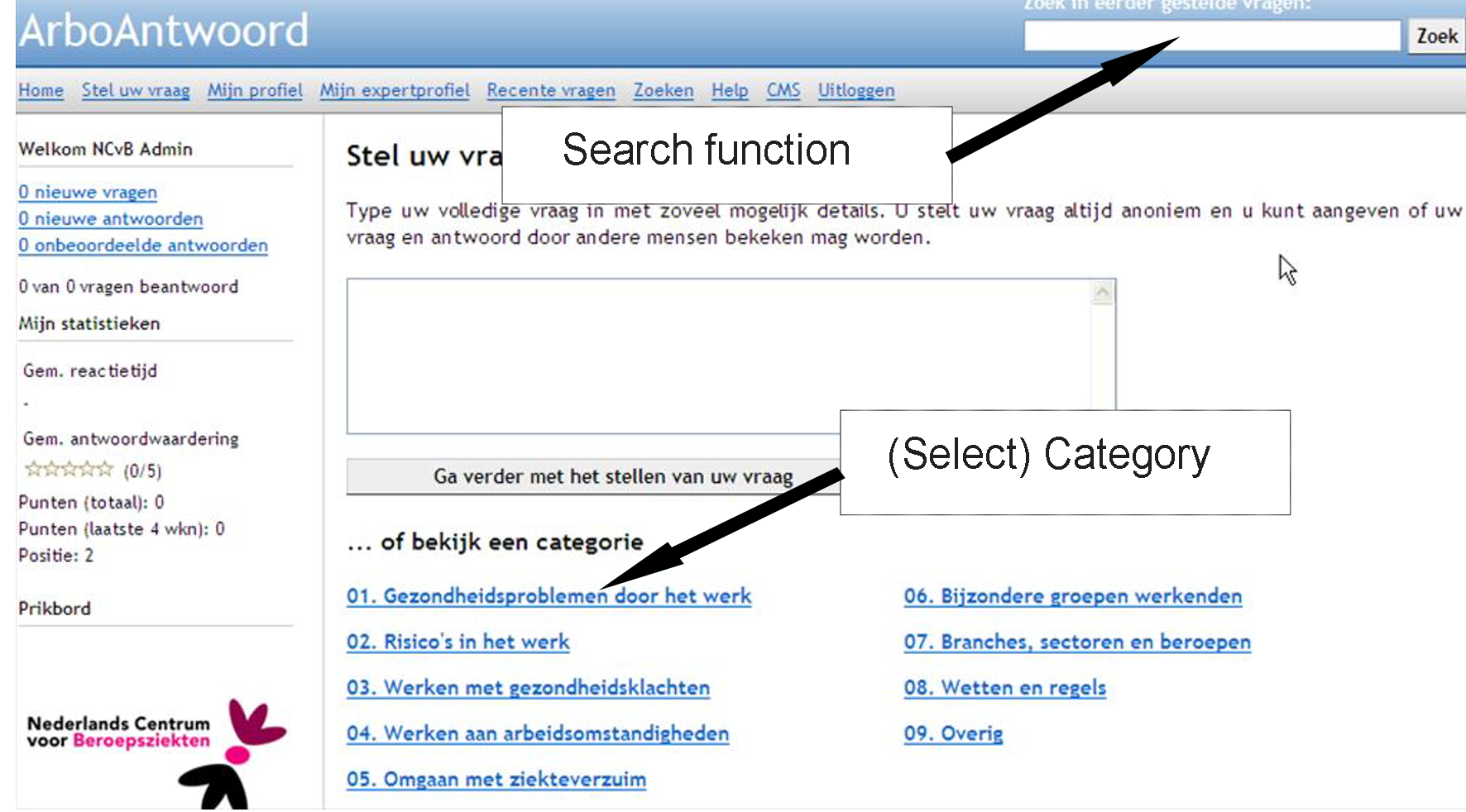

Supplement: Additional file 1 — Select category - Search function. [file 1472-6947-10-63-S1.TIFF]

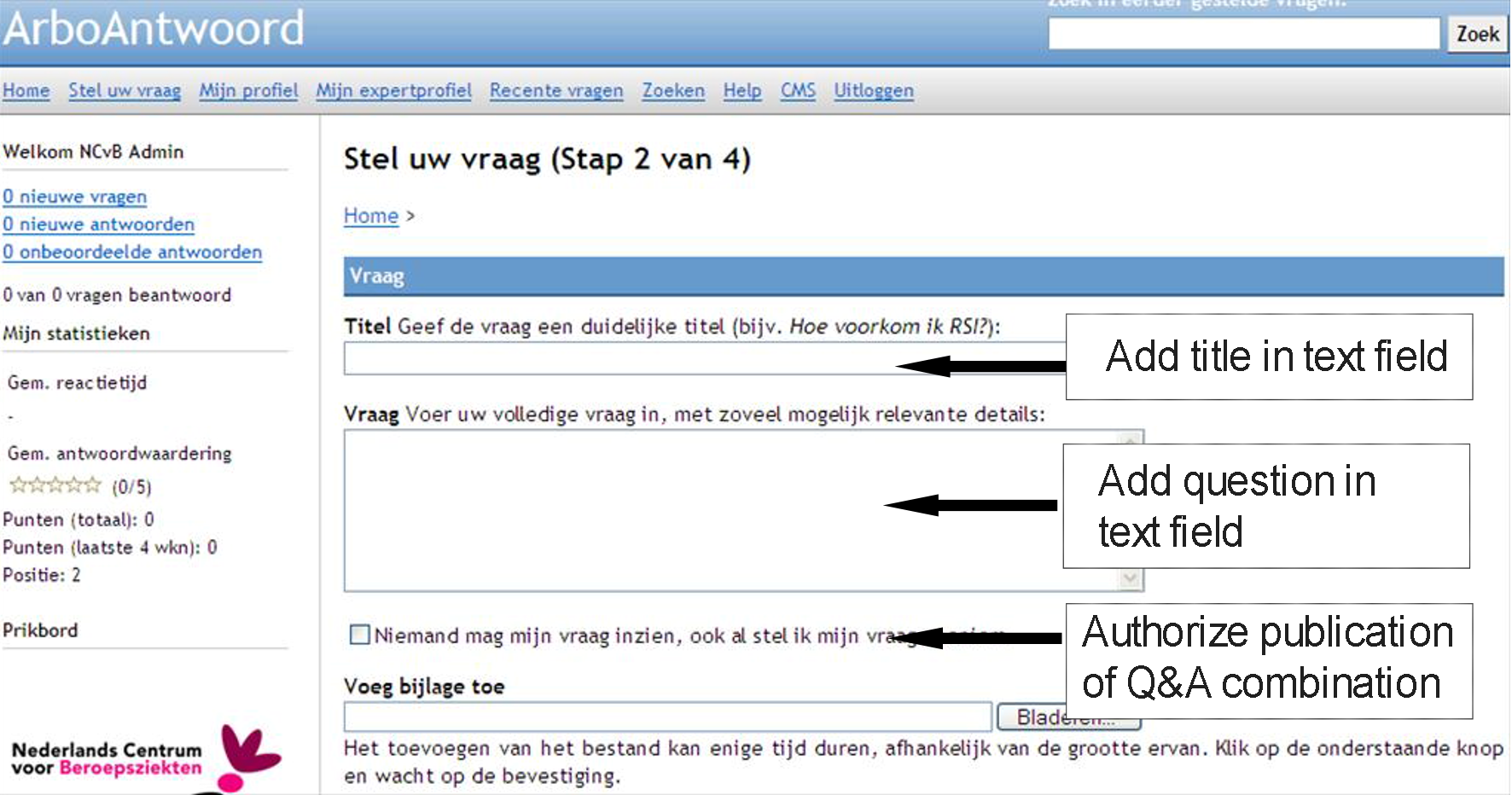

Supplement: Additional file 2 — Add question - Authorise publication. [file 1472-6947-10-63-S2.TIFF]

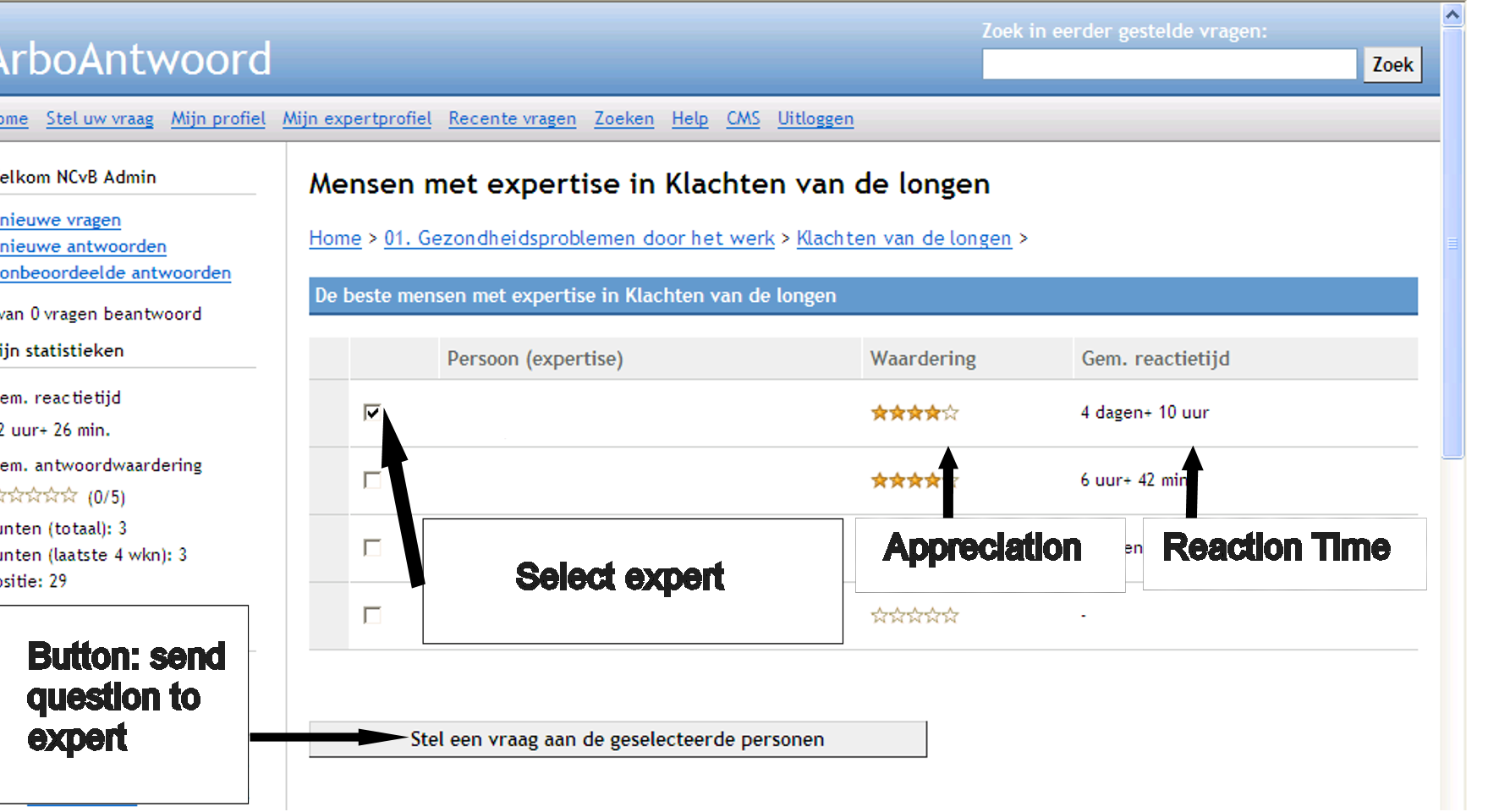

Supplement: Additional file 3 — Select expert(s) - Button to send question - Expert reaction time and appreciation. [file 1472-6947-10-63-S3.TIFF]

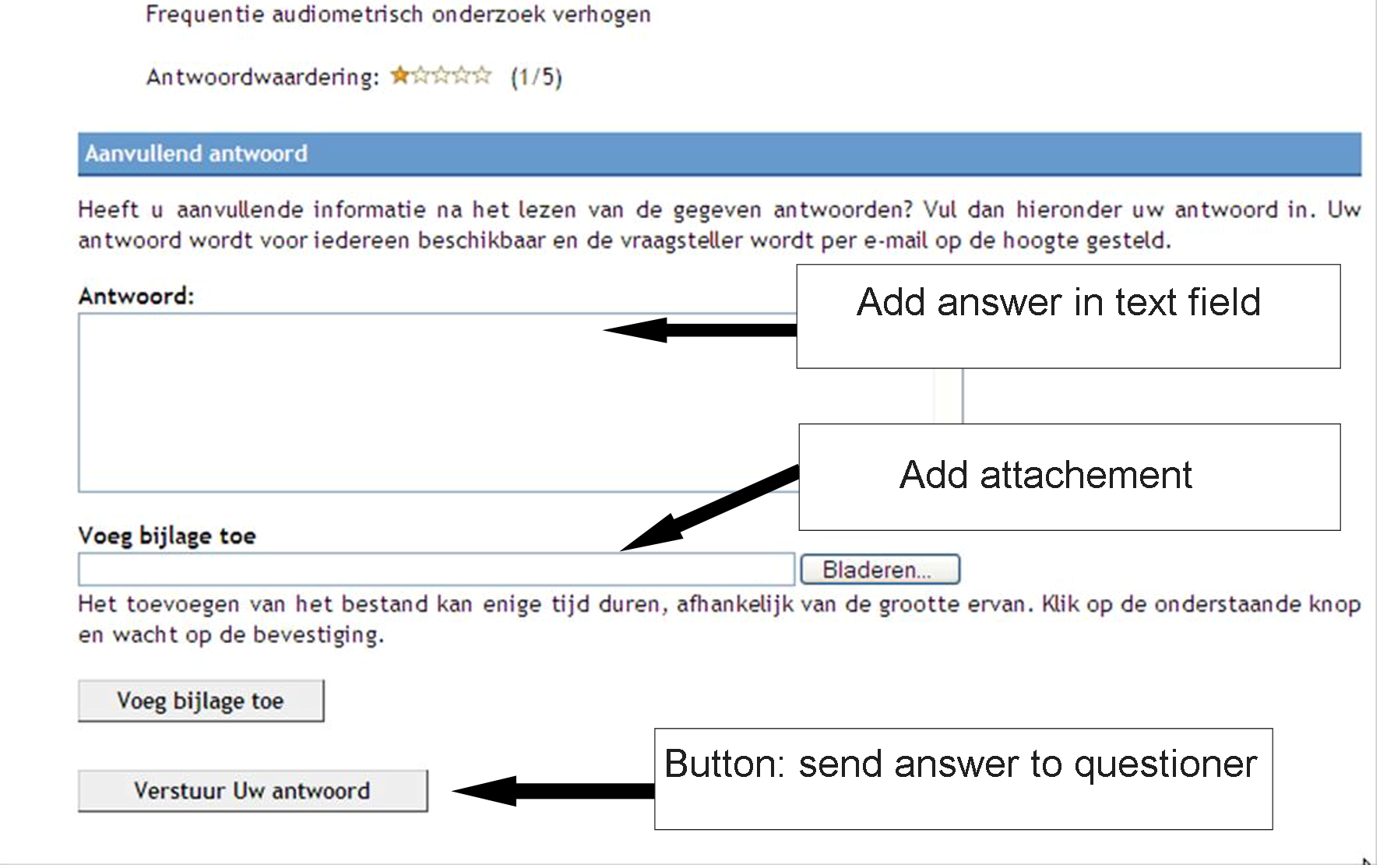

Supplement: Additional file 4 — Add answer in text field - Add attachment (optional) - Button to send answer [file 1472-6947-10-63-S4.TIFF]

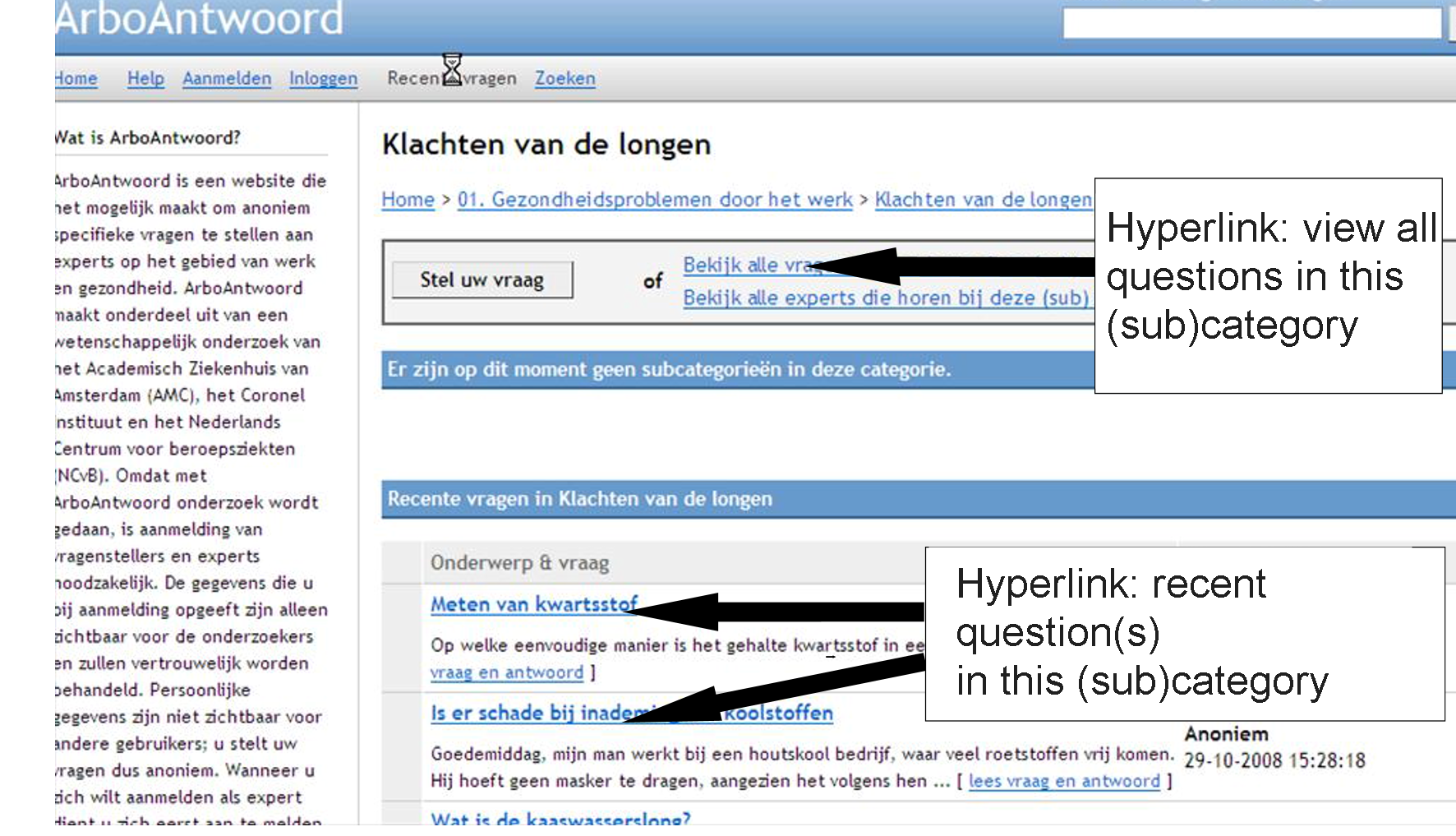

Supplement: Additional file 5 — Hyperlink to view stored Q&A - Recent Q&A in (sub)category. [file 1472-6947-10-63-S5.TIFF]
