# Supplementary material for: An online network tool for quality information to answer questions about occupational safety and health: usability and applicability
Source: BMC Med Inform Decis Mak. 2010 Oct 22;10:63. doi: 10.1186/1472-6947-10-63 (PMC2987966; doi:10.1186/1472-6947-10-63)
Supplement: Additional file 6 — Overview of all statements about facilitators, barriers and improvements of all eight features mentioned by the participants during task interviews. Participants (N = 20) are questioners from the working population (N = 12) and experts (N = 8). Questioners executed Tasks 1, 2, 3, 4 and 5. Experts executed Tasks 1, 4, 6, 7 and 8. [file 1472-6947-10-63-S6.DOC]

| **Task/Feature N** | **Facilitators** | **n** | **Barriers/ solutions** | **n** |
| --- | --- | --- | --- | --- |
| *Task 1* 20  *Register* | Amount and type of personal information is same as usual  Staying logged in is useful for next visit (cookie) | 10  5 | URL does not correspond with name website: limits access  Offer description for required personal registration information and show obligatory information: quantity, numbers and digits | 11  8 |
| *Task 2* 12  *Ask a question* | Navigation from screen to screen is made easy because there was a predefined pathway to one single end point : question + title 🡪 choose category 🡪 choose expert | 4 | Navigation can be improved: add (tracking) steps in questioning process  Questioner should select expert, not computer  Decrease scrolling in screens | 6  4  3 |
| *Task 3* 12  *Search answer in search function* | Search function website in right upper corner  Search function that indexes words (parts), combinations of words or sentences is an important feature | 5  3 | How does the search function work? Explain the search function directly above, or in help function | 4 |
| *Task 4* 20  *Search answer by category* | Clear that additional reactions are placed chronologically under primary Q&A combination  Pleasant that a second method for searching stored answers exists | 4  3 | State logic categories (alphabetic, chronologic, organ system/ risk factors, target group)  Unambiguous categories are necessary | 15  7 |
| *Task 5* 12  *Technical help &*  *help function* | Explaining movies in help function is pleasant, because it facilitates understanding of the technical problem  Presentation of hyperlinks as questions, like windows, is pleasant | 9  4 | Help function is unfindable: place in header  Place some but not too many hyperlinks in help function: categorise technical questions  Search function must index help questions (subjects) | 7  4  3 |
| *Task 6* 8  *Register as expert* | Presentation of detailed information about expert registration process was pleasant (add possibly in help function) | 8 | Register twice is illogical: register only once  Separate expert and questioner part of websites: two entrances  Who is an expert?: define rules | 8  5  3 |
| *Task 7* 8  *Answer a question*  *(n=8)* | Email notification with link directly to question safes time  Possibility to add supplement is an additional value for questioners and saves time typing  Saving draft answers is important: you do not lose it when interruptions or technical problems occur | 4  4  3 | Who is the questioner?: for a good answer, experts need function and age of questioner | 4 |
| *Task 8*  8  *Add a supplementary answer* | Presence of a supplementary answer button in same screen as stored Q&A combination is easy to use | 3 | Keep (supplementary) questions and (supplementary) answers together  Create fold-in-fold-out mechanism of answers and reactions, similar as used in Gmail or many patient forums so it will not get too complicated | 6  3 |
